# Healthy school, healthy teachers: mediating effect of optimism

**DOI:** 10.3389/fpsyg.2025.1506161

**Published:** 2025-05-22

**Authors:** Liberata Borralho, Adelinda Araújo Candeias, Saúl Neves de Jesus, João Viseu

**Affiliations:** ^1^Research Center in Education and Psychology, University of Evora, Évora, Portugal; ^2^University Center for Research in Psychology, University of Algarve, Faro, Portugal; ^3^Research Centre for Tourism, Sustainability and Well-being, University of Algarve, Faro, Portugal; ^4^Comprehensive Health Research Center, University of Evora, Faro, Portugal

**Keywords:** occupational health, optimism, organizational health, teacher’s health, teacher’s work risks, professional wellbeing

## Abstract

**Purpose:**

In recent decades, we have witnessed a growing deterioration in teachers’ health and wellbeing, which affects the quality of the teaching and learning process and the school as an organization. The school must provide a quality service that ensures student success. For this, it is essential that teachers feel healthy, satisfied, competent, and active in their work environment, maintaining wellbeing, energy, and appreciative relationships. Organizational and personal variables related to positive psychology have been scarcely studied in educational research concerning teachers’ health.

**Methodology:**

This study aimed to understand: (a) the direct relationships between organizational health and the various dimensions of teachers’ health (professional wellbeing, exhaustion, and cognitive, musculoskeletal, and voice disorders); (b) the direct relationship between organizational health and optimism; (c) the indirect effects of optimism on the relationship between organizational health and the various dimensions of teachers’ health. The research protocol was applied online to a sample of 12,104 Portuguese teachers from basic and secondary education. To analyze the data, the mediation model of organizational health on teachers’ health was evaluated using structural equation modeling (SEM), considering the mediating effect of optimism across the entire sample.

**Findings:**

The results confirmed the tested hypotheses. Organizational health is positively associated with optimism, professional wellbeing and negatively associated with exhaustion, cognitive disorders, musculoskeletal disorders, and voice changes. Similarly, optimism shows a positive relationship with professional wellbeing while being negatively linked to exhaustion, cognitive disorders, musculoskeletal disorders, and voice changes. Optimism mediates the relationship between organizational health and the various dimensions of teachers’ health.

**Conclusion:**

This study highlights the importance of organizational health in teachers’ health, emphasizing the mediating role of optimism in reducing the negative impacts of school organization on various dimensions of teachers’ health.

## 1 Introduction

In recent years, the relationship between teachers’ health and organizational health in schools has attracted growing interest in educational research. The studies by Borralho et al. (2020) and Bagdziuniene et al. (2023) indicate that a positive organizational environment—characterized by a supportive climate, effective leadership, and strong interpersonal relationships—has a significant impact on teachers’ physical and mental health, influencing aspects such as professional wellbeing, exhaustion, cognitive disorders, musculoskeletal issues, and voice alterations. Research also highlights that fostering a positive organizational culture through interventions aimed at improving teachers’ well-being can lead to enhanced job satisfaction and resilience, ultimately benefiting both educators and students (Van Woerkom, 2021).

Organizational health refers to an organization’s ability to maintain high levels of adaptability and flexibility when responding to external demands, promoting strong integration and satisfaction among its members (Gomide Júnior et al., 1999; Gomide Júnior and Fernandes, 2008; Hoy and Feldman, 1987). In schools with robust organizational health, effective management and healthy interpersonal relationships reduce stress and burnout while enhancing job satisfaction and creating an environment conducive to fostering teacher optimism (Ávalos-González and Reyes, 2022; Skaalvik and Skaalvik, 2020). Studies have also suggested that when schools adopt structured strategies for promoting organizational health, teachers experience higher levels of well-being and professional engagement (Laranjeira and Querido, 2022). On the other hand, in schools where organizational health is lacking, teachers’ optimism can act as a resilience factor, mitigating the negative effects of adverse environments and reducing the risk of burnout (Merino-Tejedor et al., 2020).

The World Health Organization (1995) defines a healthy school as one that promotes the physical, mental, and social wellbeing of all members, while fostering a safe and inclusive learning environment. A holistic focus on health, combined with strong organizational health and optimism, creates favorable working conditions for teachers, preventing health problems commonly associated with stressful work environments (Luthans et al., 2007; Seligman, 1998). Rodríguez-Mantilla and Fernández-Díaz (2019) further emphasize that organizational support and positive leadership are critical elements in reducing work-related stress among teachers, contributing to both individual and institutional well-being.

Salanova (2008) extends this concept of a “healthy organization” by highlighting the importance of developing positive psychological capital—such as optimism, resilience, and hope—among teachers as strategies for adapting to occupational stress and preventing burnout. A positive organizational environment that prioritizes teachers’ psychological wellbeing and encourages a culture of mutual respect directly contributes to teaching quality and teacher retention (Omoyemiju and Adediwura, 2011).

Recent studies also suggest that promoting optimism within organizational contexts not only improves individual wellbeing but also cultivates a supportive organizational culture, enhancing employee retention and overall performance (Dextras-Gauthier et al., 2023). As a psychological resource, optimism fosters resilience and more adaptive coping strategies, promoting a proactive approach to workplace challenges and improving overall organizational outcomes (Luthans et al., 2007; Carver and Scheier, 2014).

Moreover, optimism has been shown to play a mediating role between organizational health and teachers’ wellbeing, helping mitigate the effects of less favorable organizational environments (Song, 2022; Luthans and Youssef-Morgan, 2017). This perspective is supported by Positive Psychology and Proactive Coping models, which suggest that optimistic individuals view challenges as temporary and specific, allowing for better problem-solving and adaptation (Seligman, 1998; Peterson, 2000). In educational settings, teachers with higher levels of optimism are more likely to adopt effective coping strategies, resulting in lower stress and burnout rates, and fewer health issues related to the profession (Merino-Tejedor et al., 2020; Skaalvik and Skaalvik, 2020).

Luthans et al. (2007) include optimism within the concept of Positive Psychological Capital (PsyCap), which, alongside hope, resilience, and self-efficacy, constitutes a set of resources that support wellbeing and professional performance. In schools, this translates into healthier work environments and greater job satisfaction among teachers, ultimately enhancing teaching quality (Luthans and Youssef-Morgan, 2017; Heffernan et al., 2021).

Overall, research indicates that optimism not only protects against burnout but also contributes to the overall wellbeing of teachers, which directly impacts their physical and mental health, and subsequently, student outcomes (Bagdziuniene et al., 2023; Peterson, 2000). In challenging organizational environments, optimism is an essential tool for enhancing teachers’ health, acting as a mediator between organizational health and various dimensions of wellbeing (Kuo, 2022). The existing literature highlights the importance of promoting both optimism and organizational health as a strategy to enhance teachers’ wellbeing and create positive learning environments. However, despite previous research on these relationships, there remains a gap in understanding how optimism specifically mediates the impact of organizational health on different dimensions of teachers’ health, particularly in large-scale studies. This study addresses this gap by analyzing a substantial sample of Portuguese school teachers, offering a more comprehensive perspective on the interplay between organizational health, optimism, and teacher wellbeing, thereby contributing to the development of targeted interventions for improving teachers’ professional health and performance.

**Figure 1:**
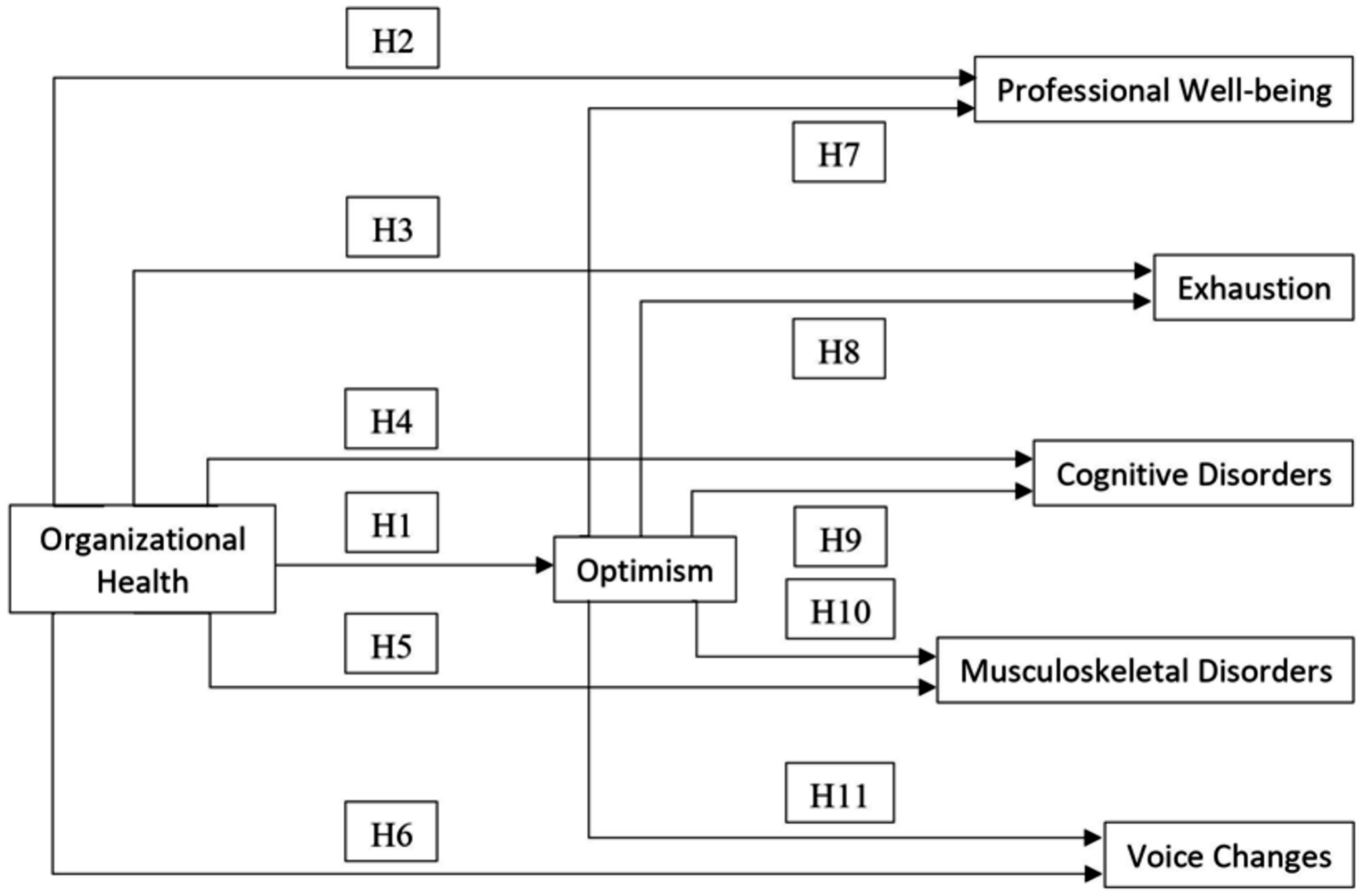
Theoretical model tested and research hypotheses proposed.

Based on the above, we present in Figure 1 the theoretical model and the corresponding hypotheses of this study.

## 2 Method

This study employed a cross-sectional design using a quantitative methodology (Montero and León, 2007). This approach, which utilizes tools such as surveys and statistical analyses, was chosen to assess the impact of organizational health and optimism on various dimensions of teachers’ health, including professional wellbeing and cognitive disorders. The methodology enhances the reliability and generalizability of the findings, providing robust evidence on the relationship between organizational factors and health outcomes.

### 2.1 Participants

A total 12,104 Portuguese teachers (≅ 9% of population), from elementary and high schools, answered the research protocol. Of these, 79.9% (*n* = 9,423) were females and 22.1% (*n* = 2,681) were males. Most teachers work in the public sector (*n* = 11,422; 94.4%). According to the defined age groups, we observed that the majority of teachers were included in the group between 46–55 years old (*n* = 5,207; 43%), with an average age of 49 years (*M* = 49.1; SD = 7.7). The most common educational background level of our sample was the bachelor’s degree. Most teachers have more than 20 years of teaching experience (*n* = 7,843; 64.8%), with an average age of 24 years (*M* = 24.1; SD = 8.8).

### 2.2 Procedures

The study was submitted to the General Directorate of Education (registration number: 057300002). The research protocol was converted into an electronic format using the Limesurvey software, following the Guidelines for E-Surveys (CHERRIES) (Eysenbach, 2012). After an initial electronic testing phase, the data collection protocol was applied online to a small sample of teachers to assess comprehension and feasibility. Subsequently, the protocol was disseminated to the target population via email, reaching all primary and secondary schools in Portugal. Participation was voluntary and based on informed consent. Participants were provided with detailed information regarding the study’s nature and objectives, as well as data confidentiality and anonymity assurances. A total of 12,178 questionnaires were obtained, of which 74 (0.61%) were excluded due to illegible data.

### 2.3 Tools

A sociodemographic questionnaire was used to collect information on age, gender, academic background, sector of activity, and teaching experience.

To assess teachers’ perceptions of organizational health, the “Organizational Health Perception Scale (OHPS)” (Gomide-Júnior and Fernandes, 2008; Jesus et al., 2016) was used. This scale, comprising 22 items is rated on a five-point Likert scale (1 – Totally disagree; 5 – Totally agree) and evaluates two dimensions: integration of individuals and groups (18 items) (e.g., “In my organization, individuals know the objectives that must be achieved”), and flexibility and adaptability to external demands (8 items) (e.g., “In my organization, the policies are flexible and can quickly adapt to changing needs”).

Teachers’ health status was measured using the “Teacher’s Health Questionnaire (THQ)” (Borralho et al., 2020), which includes 21 items that assess positive teaching experiences and physical and psychological symptoms associated with occupational risks. The THQ comprises five subscales: (a) Professional wellbeing (10 items) (e.g., “I am satisfied with my participation in the school”), (b) Exhaustion (3 items) (e.g., “After a workday, I feel drained”), (c) Cognitive disorders (3 items) (e.g., “Lately, I have been experiencing memory loss”), (d) Voice changes (2 items) (e.g., “My voice gets tired easily”), and (e) Musculoskeletal disorders (3 items) (e.g., “My back suffers due to the activity I do”), each rated on a five-point Likert scale (1–Never; 5–Almost Always).

Additionally, the “Optimism Scale (OS)” (Barros, 1998) was used to evaluate participants’ expectations for future outcomes. This scale includes four items (e.g., “I face the future with optimism”), rated on a five-point Likert scale (1 – Totally disagree; 5 – Totally agree).

Previous studies with adult populations (Borralho et al., 2020, 2024; Barros, 1998; Jesus et al., 2016; Fernández-Puig, 2015; Sampaio et al., 2021) reported good levels of internal consistency for the scales used. The Optimism Scale presented *α* = 0.84, the Organizational Health Scale α = 0.97 (Jesus et al., 2016) and α = 0.92 (Borralho et al., in press), while the Teacher Health Questionnaire recorded values ranging from α = 0.92 (Borralho et al., 2020) and α = 0.89 (Sampaio et al., 2021) to α = 0.71–0.87 (Fernández-Puig, 2015). Factor analyses confirmed the validity of these instruments.

### 2.4 Statistical procedures

We first evaluated the multivariate normal distribution of the sample. As shown in Supplementary Table 2, no variable exhibited skewness (*Sk*) or kurtosis (*Ku*) values indicating severe violations of normal distribution (|*Sk*| < 3 and |*Ku*| < 10) (Marôco, 2014). Since the data met these requirements, structural equation modeling (SEM) was performed using the maximum likelihood estimation (MLE) method in the Analysis of Moment Structures (AMOS) software, version 25.

The overall model fit followed a two-step strategy: in the first step, the measurement model was adjusted, and in the second step, the structural model. Initially the chi-squared goodness-of-fit test was evaluated, it is expected that this test reports *p*-values above 0.05, however in some situations statistically significant values (*p* < 0.05) may occur due to this test’s sensitivity to the sample size. To suppress this limitation, we also considered the absolute, relative, and parsimony fit indices proposed by Marôco (2014): Goodness of Fit Index (GFI > 0.90), Root Mean Square Error of Approximation (RMSEA <0.10), and Standardized Root Mean Residual (SRMR <0.08); Comparative Fit Index (CFI > 0.90), Tucker-Lewis Index (TLI > 0.90); Parsimony Comparative Fit Index (PCFI >0.60) and Parsimony Normed Fit Index (PNFI >0.60) (Byrne, 2016; Marôco, 2014).

In the analysis of the structural model and, for testing the research hypotheses, was observed the signal and the significance of direct, indirect (mediation effects), and total effects assessed with bootstrap resampling as described by Marôco (2014). The effects with *p* ≤ 0.05 were considered significant.

Validity was assessed in a three-way process, factor validity (factors loadings >0.50), convergent validity (Average Variance Extracted [AVE] ≥ 0.50), and discriminant validity (comparison between the AVE values and the squared correlation values) (Fornell and Larcker, 1981; Marôco, 2014). In turn, reliability was tested by Cronbach’s Alpha and Composite Reliability (CR). Both must present values above 0.70 (Hair et al., 2009; Marôco, 2014).

## 3 Results

In Supplementary Table 1 are presented the descriptive statistics and correlation matrix for the assessed constructs. This study tested the internal consistency of each sub-factor for the latent variable and observed variable. Even though there is no consensus about the most appropriate standard to apply, the sub-factors and observed variables were found to have internal consistency because Cronbach’s *α* was equal to or greater than 0.70 (Kline, 2005). The composite reliability was high, with values ranging between 0.76 and 0.92 (Marôco, 2014). The values of Average Variance Extracted (AVE ≥ 0.50) were indicators of an adequate convergent validity (Marôco, 2014). We can verify that the discriminant validity between factors is lower than the AVE values of each of the factors (Marôco, 2014).

All variables showed significant correlations with each other. Nonetheless, Pearson’s correlation coefficients between the three latent variables of organizational health showed a moderate to high correlation when compared with other correlation coefficients. This means that the greater the knowledge of the objectives of the educational project by the teachers who constitute the various organizational structures of the school, the greater the integration of them into collaborative work teams and the greater the capacity to respond effectively to changes.

Similarly, the correlation coefficients between exhaustion and cognitive disorders, musculoskeletal disorders and voice changes revealed that the two variables are moderate correlated, as well as between musculoskeletal and cognitive disorders, and between optimism and professional well-being.

The results of the chi-squared goodness of fit test were statistically significant (*p* < 0.05), an expected aspect since this fit index is influenced by the sample size. For the remaining indices, the fit varied between a good to a very good fit (Table 1).

**Table 1:** Overall model fit indices (*N* = 12,104).

Fit indexes	Observed value	Commentary
χ^2^	24347.454 (*p* < 0.001)	*
Absolute fit		
GFI	0.941	Good fit
RMSEA	0.042	Very good fit
SRMR	0.050	Good fit
Incremental fit		
CFI	0.941	Good fit
TLI	0.937	Good fit
Parsimonious fit		
PCFI	0.881	Very good fit
PNFI	0.878	Very good fit

Modified model. * Index with high sensitivity to large samples.

Regarding factor validity, all indicators respected the assumptions defined by the literature, i.e., factor loadings above 0.50 and statistically significant. There is evidence of convergent validity, since AVE > 0.50. The two reliability indicators, Cronbach’s Alpha and CR coefficient, were above 0.70 (Supplementary Table 2). Lastly, discriminant validity was observed given that all AVE values were higher than the squared correlation values.

**Figure 2:**
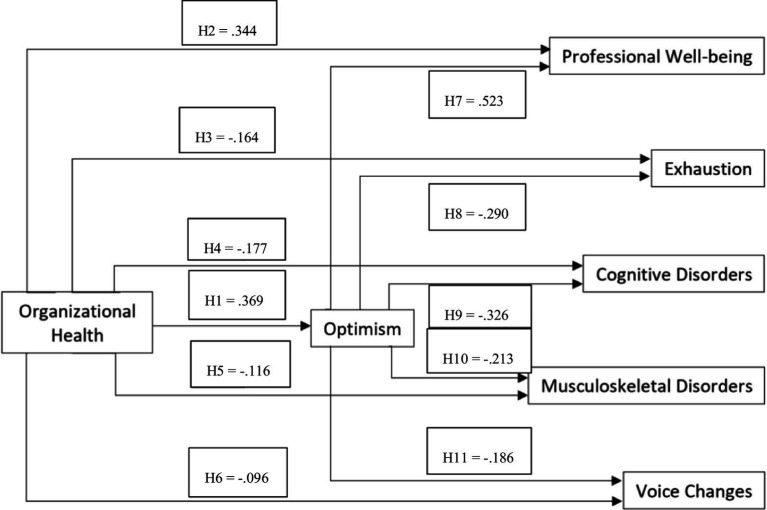
Research hypotheses results.

Relatively to H1, H2, H3, H5, and H6, which refer to the research hypotheses that tested direct relationships, it was possible to observe that: (a) organizational health was positively and significantly associated with optimism (H1), and professional wellbeing (H2); organizational health was negatively and significantly associated with Exhaustion (H3), cognitive disorders (H4), musculoskeletal disorders (H5), and voice changes (H6); (b) Thus, all these relationships were confirmed. The hypotheses concerned with the mediation effects were confirmed, given that optimism mediated the association between organizational health and professional wellbeing (H7), Exhaustion (H8), cognitive disorders (H9), musculoskeletal disorders (H10), and voice changes (H11) (Figure 2).

## 4 Discussion

The results of this study underscore the importance of organizational health in promoting teachers’ health and professional performance. The research revealed a positive and significant relationship between organizational health and both optimism and professional wellbeing, while a negative relationship was found with symptoms such as exhaustion, cognitive disorders, musculoskeletal issues, and voice changes. These findings align with previous research, which suggests that a positive institutional climate and supportive interpersonal relationships are key factors in enhancing teachers’ health and reducing the likelihood of adverse health outcomes (Borralho et al., 2020; Bagdziuniene et al., 2023; Skaalvik and Skaalvik, 2020).

A key contribution of this study is its demonstration of optimism’s mediating role in the relationship between organizational health and various dimensions of teachers’ health. While previous research has explored these relationships, this study advances existing knowledge by addressing key gaps, particularly through its large-scale sample of Portuguese teachers and the examination of variables that had not been previously investigated in this context. Optimism is confirmed as a protective factor that helps teachers navigate workplace challenges, mitigates the negative effects of weakened organizational health, and fosters overall wellbeing.

Although these findings align with prior research, this study extends the literature by offering a more comprehensive understanding of how optimism interacts with organizational health in different dimensions of teachers’ wellbeing. Specifically, its large-scale approach provides robust evidence of these relationships, reinforcing optimism as a crucial factor in effective teaching, enhanced student motivation, and the creation of positive learning environments (Sezgin and Erdogan, 2020; Heffernan et al., 2021). Furthermore, optimism is reaffirmed as a resilience factor, helping teachers maintain their professional wellbeing even in demanding school environments (Ávalos-González and Reyes, 2022; Merino-Tejedor et al., 2020).

By situating these findings within the broader context of organizational health and teacher wellbeing, this study contributes new insights that help refine theoretical models and inform practical interventions aimed at supporting educators.

However, it is important to acknowledge a potential selection bias in the sample. This study was conducted with active teachers, meaning that those who had already left the profession—possibly due to dissatisfaction, exhaustion, or difficulties in adapting to the organizational context—were not included. As a result, the findings may partially reflect a sample that overrepresents teachers with higher levels of optimism and wellbeing compared to the overall teaching population. Less optimistic teachers may be more prone to premature departure from the profession, which could influence the strength of the observed relationships.

To better understand this phenomenon, future research should employ longitudinal studies to track teachers throughout their professional careers, including those who choose to leave the profession. This approach would help clarify whether optimism plays a direct role in teacher retention and identify the factors that lead less optimistic teachers to exit the profession earlier.

Given that the present study closely resembles a national survey, it is essential to explore its practical implications, particularly regarding the development of concrete strategies that can benefit both teachers and students. The findings provide valuable insights for policymakers, school leaders, and education professionals, enabling the formulation of targeted policies and interventions to promote teacher wellbeing and improve organizational health in schools.

Furthermore, by considering the educational reality of the country, this study can contribute to the development of support programs and training initiatives for teachers, as well as to the creation of healthier and more sustainable school environments. Beyond its national impact, the study’s findings may also provide relevant insights for other countries, particularly those facing similar challenges in organizational health and teacher wellbeing. The strategies identified can serve as a reference for the development of international education policies, adaptable to different educational systems and institutional contexts.

Additionally, replicating this study in other countries would allow researchers to assess the robustness of the findings across different cultural and institutional settings, facilitating comparisons between educational systems and contributing to a broader understanding of the factors that influence teacher wellbeing and organizational health. In this sense, this study can serve as a model for future research, helping to inform evidence-based interventions that promote more balanced and sustainable teaching environments, both nationally and internationally.

Another limitation of this study is its cross-sectional design, which prevents establishing causal inferences. Additionally, the exclusive focus on Portuguese teachers may limit the generalization of findings to other cultural and institutional contexts. To address these limitations, future research should consider using longitudinal designs and exploring other potential mediators, such as resilience or emotional intelligence, to gain a more comprehensive understanding of how organizational health influences teacher wellbeing in diverse educational settings.

Future investigations should also seek to replicate the model in different cultural and institutional contexts and include additional factors that may play a role in this dynamic. Such studies could help to confirm the robustness of the findings and provide insights into how interventions targeting optimism and organizational health can be tailored to support teachers in varying environments.

Future research could explore differences in the mediating effect of optimism among teacher groups, considering age, gender, and education level. Although this study examined the overall relationship between organizational health, optimism, and teacher’s health, these variables may have a different impact on younger and more experienced teachers, men and women, or teachers in basic and secondary education. Future studies could further investigate these specificities, allowing for more targeted interventions and a broader understanding of the factors influencing teacher’s health and wellbeing.

Additionally, factors such as monthly salary, workload, and other working conditions may influence the results, representing a limitation of the study that should be considered in future research.

Beyond its academic contributions, this study also has important practical implications for schools and policymakers. The findings highlight the need for school leaders to foster a positive organizational environment that promotes optimism and wellbeing among teachers. Policies and interventions aimed at strengthening organizational health—such as providing professional support, improving leadership practices, and encouraging collaborative relationships—could help mitigate the negative effects of workplace stressors. Furthermore, these results can inform the development of evidence-based strategies to support teachers’ mental and physical health, ultimately benefiting both educators and student learning outcomes.

By emphasizing the broader value of these findings, this study contributes to the ongoing conversation on how to create healthier, more sustainable work environments for teachers.

## Data Availability

The original contributions presented in the study are included in the article/Supplementary material, further inquiries can be directed to the corresponding author.

## References

[ref1] Ávalos-González G. Reyes M. (2022). Optimism and teacher well-being: a coping strategy in challenging school environments. Educ. Psychol. Rev. 34, 123–139. doi: 10.1007/s10648-021-09569-3

[ref2] Bagdziuniene D. Liik K. Petrauskiene R. (2023). Emotional resilience and teacher well-being: the role of job and personal resources. Front. Psychol. 14:1188376. doi: 10.3389/fpsyg.2023.1188376

[ref3] Barros J. (1998). Optimismo: Teoria e avaliação (proposta de uma nova escala). Psicol. Educaç. Cult. 11, 1011–1017.

[ref4] Borralho L. Candeias A. Viseu J. Neves S. (in press). Towards a model for school organizational health: construct validation and analysis of teachers’ perceptions in Portugal. Bionet J. Biocentric Sci. 7.

[ref5] Borralho L. Jesus S. N. Viseu J. Candeias A. (2020). Avaliação da saúde dos professores portugueses: O Questionário de Saúde Docente. Psicologia 34, 195–213. doi: 10.17575/psicologia.v34i1.1475

[ref6] Byrne B. (2016). Structural equation modeling with AMOS: Basic concepts, application, and programing.(3^rd^ ed.). New York, NY: Routledge.

[ref7] Carr A. Cullen K. Keeney C. Canning C. Mooney O. Chinseallaigh E.. (2020). Effectiveness of positive psychology interventions: a systematic review and meta-analysis. J. Posit. Psychol. 15, 591–603. doi: 10.1080/17439760.2019.1651901

[ref9001] Carver C. S. Scheier M. F. (2014). Perspectives on personality. 7^th^ Edn. Boston, MA: Pearson.

[ref9002] Dextras-Gauthier J. Marchand A. Haines V. Y. I. I. I. (2023). Organizational culture and leadership behaviors: Is manager’s psychological health a missing link? Front. psychol. 14:1237775. doi: 10.3389/fpsyg.2023.1237775 37842699 PMC10569222

[ref8] Donaldson S. I. Lee J. Y. Donaldson S. I. (2019). Evaluating positive psychology interventions in the workplace: a meta-analytic review. J. Occup. Health Psychol. 24, 387–409. doi: 10.1037/ocp0000135, PMID: 30335420

[ref9] Eysenbach G. (2012). Correction: improving the quality of web surveys: the checklist for reporting results of internet E-surveys (CHERRIES). J. Med. Internet Res. 14:e8. doi: 10.2196/jmir.2042 PMC1550605 15471760

[ref10] Fernández-Puig V. Longás Mayayo J. Chamarro Lusar A. Virgili Tejedor C. (2015). Evaluando la salud laboral de los docentes de centros concertados: El Cuestionario de Salud Docente. J. Work Organ. Psychol. 31, 175–185. doi: 10.1016/j.rpto.2015.07.001

[ref11] Fornell C. Larcker D. (1981). Evaluating structural equation models with unobservable variables and measurement error. J. Mark. Res. 18, 39–50. doi: 10.2307/3151312

[ref13] Gomide Júnior S. Fernandes M. (2008). Saúde organizacional. In Siqueira M. et al., Medidas do comportamento organizacional: Ferramentas de diagnóstico e de gestão (pp. 275–282). Porto Alegre, Brasil: Artmed.

[ref14] Gomide Júnior S. Moura M. A. P. Cunha E. T. Sousa D. F. (1999). Saúde organizacional: Uma abordagem integrada: Mesquita, RJ: Editora XYZ.

[ref15] Hair J. Black W. Babin B. Anderson R. (2009). Multivariate data analysis. (7^th^ ed.). Upper Saddle River, NJ: Pearson Prentice Hall.

[ref16] Heffernan A. Fogarty G. Richards J. (2021). Teacher optimism and resilience in challenging school environments. J. Educ. Psychol. 112, 466–493. doi: 10.1037/edu0000422, PMID: 39780972

[ref9003] Hoy W. K. Feldman J. A. (1987). Organizational health: The concept and its measure. J. Res. Dev. Educ. 20, 30–37.

[ref17] Jesus S. N. Viseu J. Lobo P. Orgambídez-Ramos A. Moura D. Santos J.. (2016). “Validação de uma medida de saúde organizacional para a população portuguesa.” In Chambel M. J. (Ed.), Psicologia da saúde ocupacional (pp. 51–69). Lisboa, Portugal: Editora Pactor.

[ref18] Kline R. B. (2005). Principles and practice of structural equation modeling. 2nd Edn. New York, NY: Guilford Press.

[ref19] Kuo S. (2022). Well-being of teachers: the role of efficacy of teachers and academic optimism. Front. Psychol. 12:831972. doi: 10.3389/fpsyg.2021.831972, PMID: 35153944 PMC8825852

[ref20] Laranjeira C. Querido A. (2022). Hope and optimism as an opportunity to improve the “positive mental health” demand. Front. Psychol. 13:827320. doi: 10.3389/fpsyg.2022.827320, PMID: 35282230 PMC8907849

[ref9004] Luthans F. Avolio B. J. Avey J. B. Norman S. M. (2007). Positive psychological capital: Measurement and relationship with performance and satisfaction. Pers. Psychol. 60, 541–572. doi: 10.1111/j.1744-6570.2007.00083.x

[ref9005] Luthans F. Youssef-Morgan C. M. (2017). Psychological capital: An evidence-based positive approach. Annu. Rev. Organ. Psych. 4, 339–366. doi: 10.1146/annurevorgpsych-032516-113324

[ref21] Marôco J. (2014). Análise de equações estruturais: Fundamentos teóricos, software e aplicações. 2nd Edn (2^nd^ ed.). Pêro Pinheiro, Portugal: ReportNumber.

[ref22] Merino-Tejedor E. Hontangas P. M. Boada-Grau J. (2020). Optimism as a mediating factor in the stress-burnout relationship. Psychol. Rep. 123, 71–96. doi: 10.1177/0033294119852574, PMID: 31142189

[ref9006] Montero I. León O. (2007). A guide for naming research studies in Psychology. Int. J. Clin. Health Psychol. 7, 847–862.

[ref9007] Omoyemiju M. A. Adediwura A. A. (2011). A study of teachers’ perception of schools’ organizational health in Osun state. World J. Educ. 1, 165–170. doi: 10.5430/wje.v1n1p165

[ref9008] Peterson C. (2000). The future of optimism. Am Psychol. 55, 44–55. doi: 10.1037/0003-066X.55.1.44 11392864

[ref23] Rodríguez-Mantilla J. M. Fernández-Díaz M. J. (2019). Organizational commitment, group cohesion, and their influence on teacher well-being. Educ. Res. 61, 343–359. doi: 10.1080/00131881.2019.1657167

[ref9009] Salanova M. (2008). “Organizaciones saludables y desarrollo de recursos humanos” in Organizaciones saludables: Una mirada desde la Psicología Positiva. ed. Salanova M. (Barcelona, España: Aedipe), 15–38.

[ref24] Sampaio A. A. Stobaus C. D. Lima D. F. Mazzardo O. Piovani V. G. S. Both J. (2021). Validação do Questionário Saúde Docente para o contexto brasileiro. J. Phys. Educ. 32:e3228. doi: 10.4025/jphyseduc.v32i1.3228, PMID: 23108262

[ref9010] Seligman M. E. P. (1998). Learned optimism: How to change your mind and your life. New York, NY: Pocket Books.

[ref9011] Sezgin F. Erdogan O. (2020). The relationship between teacher psychological capital and job satisfaction: A structural equation modeling approach. Int. J. Educ. Methodol. 6, 1–12. doi: 10.12973/ijem.6.1.1

[ref25] Skaalvik E. M. Skaalvik S. (2020). Teacher burnout: relations between organizational health and emotional exhaustion. Teach. Teach. Educ. 87:102935. doi: 10.1016/j.tate.2019.102935, PMID: 40088920

[ref9012] Song Z. (2022). Optimism and teacher burnout: The mediating role of psychological resilience. Front. Psychol. 13:865432. doi: 10.3389/fpsyg.2022.865432

[ref26] Van Woerkom M. (2021). Building positive organizations: a typology of positive psychology interventions. Front. Psychol. 12:769782. doi: 10.3389/fpsyg.2021.769782, PMID: 34867675 PMC8637171

[ref27] World Health Organization (1995). Promoting health through schools: The WHO’s global school health initiative. Geneva, Switzerland: World Health Organization.

