# Supplementary material for: Healthy school, healthy teachers: mediating effect of optimism
Source: Front Psychol. 2025 May 22;16:1506161. doi: 10.3389/fpsyg.2025.1506161 (PMC12140021; doi:10.3389/fpsyg.2025.1506161)
Supplement: Supplementary file 1 [file Supplementary_file_1.docx]

Supplementary Material

Table 1. Descriptive Statistics and Correlation Matrix (N = 12104)

| Constructs | *M* | *SD* | 1. | 2. | 3. | 4. | 5. | 6. | 7. | 8. | 9. |
| --- | --- | --- | --- | --- | --- | --- | --- | --- | --- | --- | --- |
| 1. Integration of people and teams | 3.20 | 6.00 | - |  |  |  |  |  |  |  |  |
| 2. Flexibility and adaptability to external requirements | 2.93 | 6.43 | **.711** | - |  |  |  |  |  |  |  |
| 3. Knowledge of objectives | 3.33 | 5.71 | **.804** | **.743** | - |  |  |  |  |  |  |
| 4. Professional well-being | 2.25 | 6.59 | .414 | .430 | .436 | - |  |  |  |  |  |
| 5. Exhaustion | 3.51 | 2.72 | -.219 | -.247 | -.220 | -.493 | - |  |  |  |  |
| 6. Cognitive disorders | 3.97 | 2.76 | -.215 | -.213 | -.212 | -.464 | **.648** | - |  |  |  |
| 7. Musculoskeletal disorders | 3.76 | 3.85 | -.172 | -.157 | -.147 | -.311 | **.585** | **.547** | - |  |  |
| 8. Voice changes | 2.86 | 2.01 | -.144 | -.150 | -.137 | -.314 | **.524** | .478 | .480 | - |  |
| 9. Optimism | 3.59 | 3.47 | .319 | .309 | .308 | **.557** | -.344 | -.351 | -.258 | -.233 | - |

Note. M = mean values; SD = standard-deviation values; All the correlation values statistically significant at a .01 level.

Table 2. Results for skewness, Kurtosis, Factor Validity, Convergent Validity, Reliability and Discriminant Validity

| Constructs and Scale Items | Sk | Ku | Std. Loading^*^ | Alpha/CR | CR | AVE |
| --- | --- | --- | --- | --- | --- | --- |
| **Organizational Health (OH) 24 items** |  |  |  | .96 |  |  |
| **Knowledge of Objectives (KO) 8 items** |  |  |  | .91 | .92 | .58 |
| OH1 - People know the objectives that the institution intends to achieve. | -.746 | .165 | .680 | .96 |  |  |
| OH2 - People work together so that the institution reaches its objectives. | -.281 | -.730 | .761 | .96 |  |  |
| OH3 - It is possible to talk about perceived problems directly with the people involved. | -.473 | -.472 | .748 | .96 |  |  |
| OH4 - People have access to the information needed to make decisions related to work. | -.658 | -.017 | .710 | .96 |  |  |
| OH5 - Actions are planned as a team. | -.529 | -.346 | .764 | .96 |  |  |
| OH6 - There is cooperation between people in carrying out the work. | -.605 | -.148 | .766 | .96 |  |  |
| OH7 - Problem solving is sought, so that all the people involved can give their opinion. | -.395 | -.528 | .828 | .96 |  |  |
| OH8 - Individual needs are considered, when it is necessary to diagnose organizational problems. | -.203 | -.659 | .760 | .96 |  |  |
| **Integration of People and Teams (IPT) 9 items** |  |  |  | .92 | .92 | .63 |
| OH9 - The competition between the teams is done honestly. | -.281 | .008 | .726 | .96 |  |  |
| OH10 - People spontaneously seek to help their colleagues through suggestions. | -.533 | -.276 | .784 | .96 |  |  |
| OH11 - People spontaneously seek to help their colleagues, through concrete actions. | -.344 | -.398 | .792 | .96 |  |  |
| OH12 - When there are crises, people come together to work cooperatively to resolve them. | -.381 | -.467 | .794 | .96 |  |  |
| OH13 - People have respect for others. | -.548 | -.133 | .783 | .96 |  |  |
| OH14 - People see their work as something important. | -.716 | .635 | .651 | .96 |  |  |
| OH16 - People trust each other. | -.271 | -.447 | .790 | .96 |  |  |
| OH18 - People accept constructive criticism of their work performance. | -.207 | -.783 | .682 | .96 |  |  |
| OH19 - People try to help their colleagues who perform poorly at work. | -.149 | -.734 | .730 | .96 |  |  |
| **Flexibility and Adaptability to External Requirements (FAER) 7 items** |  |  |  | .91 | .91 | .57 |
| OH15 - Heads vary their management styles, depending on the needs of different work situations. | -.332 | -.455 | .661 | .96 |  |  |
| OH17 - There is a general feeling of freedom. | -.210 | -.420 | .720 | .96 |  |  |
| OH20 - Policies are flexible and can adapt quickly to changing needs. | -.170 | -.463 | .816 | .96 |  |  |
| OH21 - The procedures are flexible and can adapt quickly to changing needs. | -.196 | -.526 | .844 | .96 |  |  |
| OH22 - Policies are designed to help people be effective at work. | -.026 | -.640 | .844 | .96 |  |  |
| OH23 - The procedures are established in order to help people to be effective at work. | -.047 | -.658 | .855 | .96 |  |  |
| OH24 - Innovations are continually sought. | -.048 | -.742 | .663 | .96 |  |  |
| **Teacher’s Health (TH) 21 items** |  |  |  | .92 |  |  |
| **Professional Weel-being (PWB) 9 items** |  |  |  | .91 | .92 | .52 |
| TH3 - I feel satisfied with the way I do things. | .031 | -.341 | .678 | .91 |  |  |
| TH5 - If I could, I would choose to be a teacher again. | -.384 | -.558 | .686 | .92 |  |  |
| TH9 - I enjoy my daily tasks. | -.023 | -.223 | .665 | .91 |  |  |
| TH10 - I am very happy in my work. | -.037 | -.531 | .858 | .91 |  |  |
| TH12 - When I finish a job, I am happy with the results. | -.421 | -.514 | .682 | .92 |  |  |
| TH14 - I have fun at work. | .484 | -.804 | .804 | .91 |  |  |
| TH17 - When I wake up, I feel like going to work. | -.034 | -.516 | .768 | .91 |  |  |
| TH18 - I am satisfied with my participation in the school. | .251 | -.589 | .782 | .91 |  |  |
| TH20 - I have the ability to be creative and agile in my teaching activity. | .400 | -.120 | .690 | .92 |  |  |
| **Exhaustion (EX) 3 items** |  |  |  | .88 | .81 | .70 |
| TH6 - After a day of work, I feel powerless. | .455 | -.1.115 | .816 | .92 |  |  |
| TH11 - I feel physically tired at the end of my workday. | .149 | -.340 | .847 | .91 |  |  |
| TH16 - At work, I get very tired. | .033 | -.711 | .832 | .91 |  |  |
| **Cognitive Disorders (CD) 3 items** |  |  |  | .74 | .76 | .51 |
| TH2 - I have the impression that I am obsessed with a subject. that at another time I could solve without a problem. | -.281 | -.730 | .679 | .92 |  |  |
| TH8 - At times, I lack concentration to perform tasks. | -.203 | -.659 | .782 | .91 |  |  |
| TH21 - Lately I have a lack of memory. | .281 | -1.135 | .672 | .91 |  |  |
| **Musculoskeletal Disorders (MD) 4 items** |  |  |  | .81 | .81 | .51 |
| TH1 - I have pain in the neck area. | -.746 | .165 | .733 | .92 |  |  |
| TH4 - My back hurts due to the activity I do. | .321 | -.065 | .697 | .92 |  |  |
| TH13 - I feel a pain in my neck after a day at work. | .235 | -.090 | .758 | .92 |  |  |
| TH19 - I suffer from low back pain. | -.086 | -.554 | .677 | .92 |  |  |
| **Voice Changes (VC) 2 items** |  |  |  | .84 | .85 | .72 |
| TH7 - I notice that I am aphonic (a) or dysphonic (a). | -.250 | -.568 | .841 | .92 |  |  |
| TH15 - My voice gets tired easily. | .119 | -.215 | .858 | .91 |  |  |
| **Optimism (O) 4 items** |  |  |  | .91 | .91 | .72 |
| O1 - I face the future with optimism. | -.489 | -.374 | .876 | .87 |  |  |
| O2 - I hope to get what I really want. | -.758 | .218 | .904 | .86 |  |  |
| O3 - I do projects for the future and I think I will do them. | -.649 | .145 | .842 | .88 |  |  |
| O4 - In general, I consider myself an optimistic person. | -.680 | .043 | .753 | .91 |  |  |

Note. *p = .000; Sk = Skewness; Ku = Kurtosis; Std. loading = standardized factor loading; Alpha/CR = Cronbach’s alpha; CR - Composite Reliability ; AVE = average variance extracted.
